# Drug rash with eosinophilia and systemic symptoms (DRESS) syndrome in childhood: a narrative review

**DOI:** 10.3389/fmed.2023.1108345

**Published:** 2023-07-28

**Authors:** Elisa Manieri, Arianna Dondi, Iria Neri, Marcello Lanari

**Affiliations:** ^1^Specialty School of Pediatrics, Alma Mater Studiorum, University of Bologna, Bologna, Italy; ^2^Pediatric Emergency Unit, IRCCS Azienda Ospedaliero-Universitaria di Bologna, Bologna, Italy; ^3^Division of Dermatology, IRCCS Azienda Ospedaliero-Universitaria di Bologna, Bologna, Italy

**Keywords:** children, drug reaction with eosinophilia and systemic symptoms, DRESS, drug reaction, drug hypersensitivity, antibiotics, anticonvulsants

## Abstract

Despite being rare, the Drug Rash with Eosinophilia and Systemic Symptoms (DRESS) syndrome is a serious, possibly fatal condition that may affect both adults and children who may be also burdened by delayed sequelae. It is an adverse drug reaction characterized by widespread skin involvement, fever, lymphadenopathy, visceral involvement, and laboratory abnormalities (eosinophilia, mononucleosis-like atypical lymphocytes). It is more frequently triggered by anticonvulsants, sulphonamides, or antibiotics, the latter being responsible for up to 30% of pediatric cases. The disease typically develops 2–8 weeks after exposure to the culprit medication, with fever and widespread skin eruption; mild viral prodromes are possible. Unfortunately, diagnosis is challenging due to the absence of a reliable test; however, a score by the European Registry of Severe Cutaneous Adverse Reactions (RegiSCAR) allows to classify suspect patients into no, possible, probable, or definite DRESS cases. Moreover, rapid-onset DRESS syndrome has been described in recent years. It affects children more often than adults and differs from the most common form because it appears ≤15 days vs. >15 days after starting the drug, it is usually triggered by antibiotics or iodinated contrast media rather than by anticonvulsants and has a higher presence of lymphadenopathy. Differential diagnosis between rapid-onset antibiotic-driven DRESS syndrome, viral exanthems, or other drug eruptions may be challenging, but it is mandatory to define it as early as possible to start adequate treatment and monitor possible complications. The present review reports the latest evidence about the diagnosis and treatment of pediatric DRESS syndrome.

## 1. Introduction

The Drug Rash with Eosinophilia and Systemic Symptoms (DRESS) syndrome is a rare but severe, potentially fatal, idiosyncratic, adverse drug reaction with cutaneous and systemic manifestations (1). It is characterized by widespread skin involvement with mucocutaneous and multisystem involvement, fever, lymphadenopathy, hematologic abnormalities (eosinophilia, mononucleosis-like atypical lymphocytes), and viral reactivation (2, 3); furthermore, prodromes mimicking viral infections may occur (4). The first reports of this syndrome date to the 1950s under different names, e.g., “anticonvulsant hypersensitivity syndrome,” “drug-induced hypersensitivity syndrome,” “drug-induced delayed multiorgan hypersensitivity syndrome” (5), or “drug-induced pseudolymphoma” (6), but it was Bocquet et al. (6) who first reported it in 1996 as the “DRESS syndrome.”

Although it is more common in adults, the DRESS syndrome can also affect children, resulting in long-term and delayed sequelae or even death, and effectively prohibiting patients to use possibly important drugs for life (1). Anticonvulsants (mainly phenobarbital, phenytoin, and carbamazepine), allopurinol, and sulfonamides are the most common pharmacologic triggers, but in the pediatric population, antibiotics are reported to induce up to 30% of the cases (1). Symptoms typically arise from 2 to 8 weeks after drug introduction (7), but, in children, rapid-onset cases (less than 2 weeks since the drug exposure) have been frequently reported, especially when antibiotics are involved (8). The variable clinical presentation, accounting for similarities to infectious or lymphoproliferative diseases, may delay diagnosis (8). However, early recognition may ease the start of the appropriate treatment, consisting of drug withdrawal, supportive care, and eventually the initiation of systemic corticosteroids to reduce widespread inflammation are the backbone of treatment (9, 10). These are the most significant actions to reduce disease progression.

In the present work, we report the evidence about clinical features, diagnosis, and treatment of the DRESS syndrome in children and highlight the differences between the pediatric and adult populations.

## 2. Incidence, etiology, and risk factors

The incidence of the DRESS syndrome is estimated to vary between 1 case in 1,000 and 1 case in 10,000 drug exposures. In children it is probably lower than in adults, although the exact rate is unknown (11). This syndrome is supposed to be more frequent than other severe drug-induced reactions such as Stevens-Johnson syndrome/toxic epidermal necrolysis (12), but less common than food-induced anaphylaxis. In children, the average age of occurrence of the DRESS syndrome is reported to be 9 years (11, 13) with no gender predilection (1). Neuropsychiatric disorders are the most common comorbidities, consistently with antiepileptics being the main triggers (10). In the general population, the overall mortality rate is 10%; in children, it is estimated to be around 5.4% (4, 11).

Common pharmacologic triggers for pediatric DRESS syndrome include aromatic anticonvulsants, responsible for 50% of the cases (mainly carbamazepine, phenytoin, and phenobarbital); antibiotics, responsible for up to 30% of the cases (mainly vancomycin, trimethoprim-sulfamethoxazole, amoxicillin) and, although infrequent, sulfasalazine (4.6%) and nonsteroidal anti-inflammatory drugs (4.6%) (1, 9, 14–18). In the series collected by the prospective Registry of Severe Cutaneous Adverse Reactions (RegiSCAR) study, the drugs that most frequently cause DRESS syndrome in adults are aromatic anticonvulsants, responsible for 35% of cases, followed by allopurinol in 18% of cases, sulfonamides in 12% of cases, and other antibiotics responsible for 11% of cases (19).

Anticonvulsants have a high potential for triggering the DRESS syndrome so that, although less frequently used than other drugs, are the most common cause in children and adults; on the other hand, commonly prescribed drugs like certain antibiotics have a lower potential, but in light of their more frequent prescription, they lead to a high percentage of cases.

## 3. Pathogenesis

The DRESS syndrome is sustained by a T cell-mediated delayed-type drug hypersensitivity reaction, although its complex, multifactorial pathogenesis is only partially understood (20). It combines different mechanisms, which include incomplete drug metabolism and the accumulation of reactive metabolites that can lead to a robust and delayed immunological reaction to drugs, a transient state of immunosuppression, and probably the reactivation of latent viral infections with a subsequent antiviral immune response (19). Currently, four main models potentially responsible for T-cell hypersensitivity reactions have been hypothesized: (1) the hapten/prohapten model, according to which a drug binds covalently to an endogenous protein, forming a complex, that it is processed by antigen-presenting cells and recognized by a T cell receptor (TCR), developing a drug-specific immune response; (2) the pharmacological interactions of drugs with the immune receptors, according to which the responsible drug binds directly and non-covalently to Human Leukocyte Antigens (HLA) and/or TCR, directly stimulating specific TCR and generating drug-reactive T cells; (3) the altered peptide repertoire model, according to which the culprit drug binds non-covalently to the peptide-binding pocket of an HLA, changing its conformation and allowing a new array of self-peptides to stimulate T cells; (4) the altered TCR repertoire model, according to which culprit drug binds directly to and alters specific TCRs, providing them with the ability to bind to HLA-self peptide to initiate immune responses (21).

Once these mechanisms occur, drug-specific T cells are produced.

The immune response is mainly a Th2 response, with an expansion of T cells and cytokines related to the hyper-eosinophilia, as interleukin (IL)-4, IL-5, and IL-13 and thymus and activation-regulated chemokine (TARC) (22). TARC levels have been identified as a potential biomarker of the acute phase and a predictor of disease activity in DRESS syndrome and appear to correlate with skin manifestations (21). In addition, other cytokines and chemokines which are reported to be increased in DRESS syndrome are tumor necrosis factor (TNF)-α, interferon (IFN)-γ, IL-2, and IL-6 (21). A large stimulation of T cells appears to be the decisive factor for the development of Multiple Drug Hypersensitivity (MDH), which is a syndrome characterized by drug hypersensitivity reactions to various structurally different drugs (23). A case of MDH against antibiotics in a 23-month-old girl with DRESS syndrome has recently been reported in the literature (24). This allows us to underline how MDH should be suspected in children receiving more than one drug capable of eliciting DRESS syndrome.

The reactivation of viruses of the Herpesviridae family appears to be a feature of the DRESS syndrome. Human herpesvirus (HHV)-6 is the most frequently reactivated, followed by cytomegalovirus (CMV), Epstein–Barr virus (EBV), and HHV-7 (18). The actual role, mechanisms, and timing of viral reactivation in the drug-specific immune response and DRESS syndrome pathogenesis have not been clarified yet, but there seems to be an association between it and the flaring-up of clinical symptoms (25), greater severity, or longer duration of disease (26). Viral reactivation contributes to T cell activity by inducing the synthesis of pro-inflammatory cytokines. Furthermore, the virus may participate in the drug-TCR interactions (21). One hypothesis is that viral reactivation occurs because of an immunodeficiency state; in fact, during the acute stage of the DRESS syndrome, the population of T regulatory cells is expanded, while the number of B cells and the plasma levels of immunoglobulins are reduced, which may facilitate viral reactivation (27, 28). An alternative hypothesis is that certain drugs (i.e., amoxicillin, valproic acid) may directly increase HHV-6 replication (29). Recently it has also been hypothesized that the T lymphocytes developed after exposure to the causal drug are virus-specific memory T cells, reactivated following an incorrect recognition of the HLA-drug complexes (30). The detection of HHV-6 in the peripheral blood of patients is considered a possible marker of this condition and has been included in the Japanese (31, 32), but not in the European (19, 33), diagnostic criteria, as further discussed in the related chapter.

Genetic susceptibility is thought to be linked to factors influencing immune responses. Several studies have disclosed important associations between the predisposition of hypersensitivity reactions to some drugs and specific HLA alleles (25), and since some alleles are more frequent in some ethnic groups than in others, these associations appear to be drug-, phenotype- and ethnicity-specific (25). This susceptibility could be explained by the fact that specific HLAs may have a higher binding affinity for a specific drug, eliciting adverse immune responses. For instance, some studies have highlighted an association between HLA-B*58:01, HLA*B-32:02, HLA-B*31:01, and HLA-B*13:01 and allopurinol-, vancomycin-, carbamazepine-, and dapsone-induced DRESS, respectively. It could be thus speculated that HLA may be used as an additional screening test to improve the evaluation of the likelihood of drug causality and stratify DRESS syndrome risk (34, 35).

In addition, the presence of polymorphisms in genes encoding drug-metabolizing enzymes (e.g., cytochrome P 450) may result in the accumulation of the drug or its active metabolites and have been implicated as a cofactor in the development of phenytoin-induced severe adverse cutaneous drug reactions (36).

However, these findings suggest that the syndrome’s pathogenesis is multifactorial, taking into account not only genetic susceptibility but also the presence of viral reactivation, the patient’s ethnic group, and immune status (25). These factors alone are probably not sufficient to trigger DRESS syndrome but taken together they may work synergistically to increase the risk.

**Figure 1:**
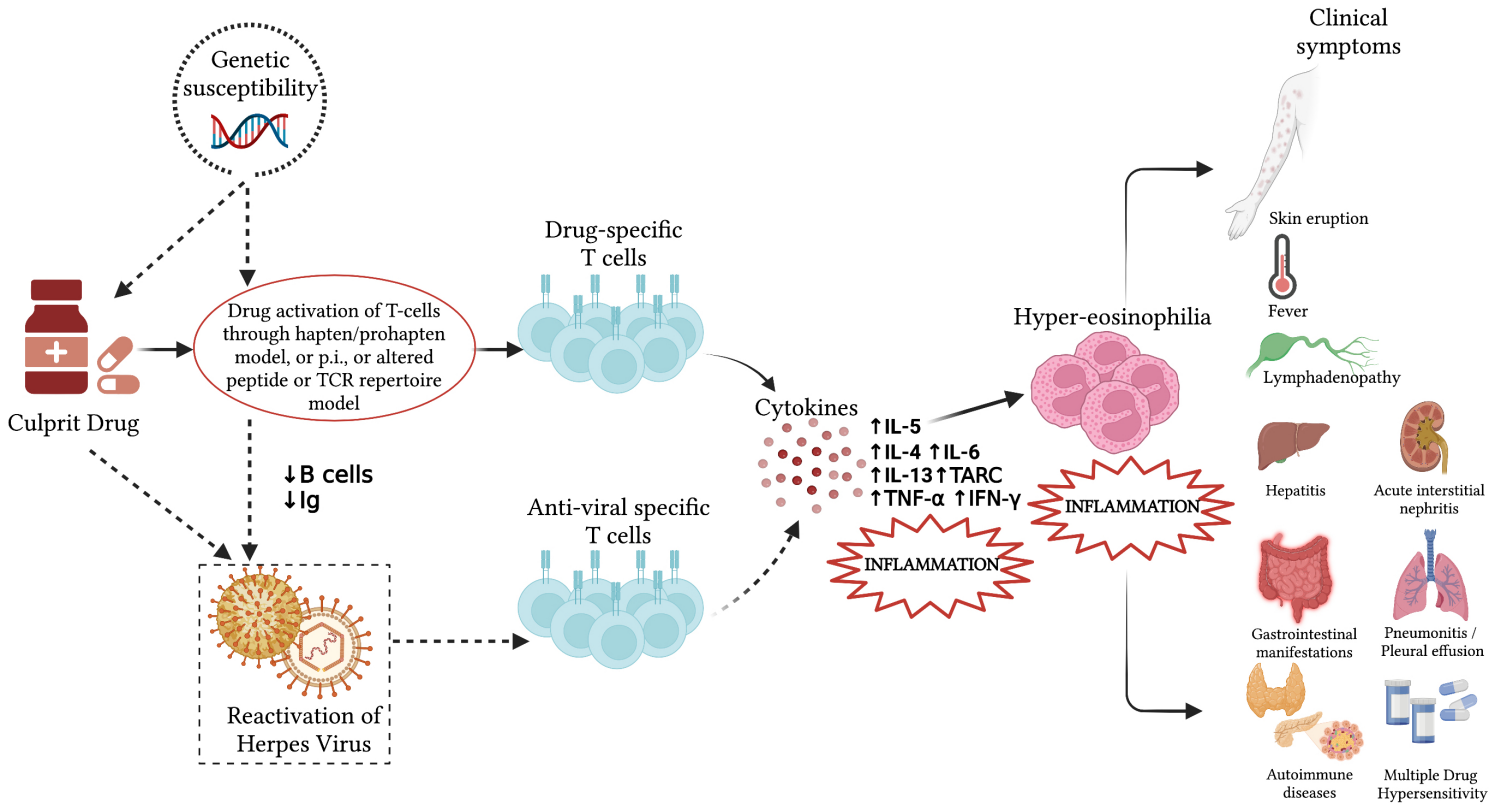
Pathogenesis of the Drug Rash with Eosinophilia and Systemic Symptoms (DRESS) syndrome. Incomplete drug metabolism and the accumulation of reactive metabolites can lead to a robust and delayed immunological reaction, stimulating drug-specific T lymphocytes in people with predisposing genetic factors and producing the systemic effects and the clinical presentation of the DRESS syndrome. In some patients, a viral reactivation may be detected between 2 and 4 weeks after symptoms onset. It results from a direct effect of the culprit drug or an immunodeficiency state caused by the anti-drug responses and may induce antiviral responses that contribute to the development of the systemic effects. The dotted line indicates possible cofactors whose exact role in the pathogenesis of DRESS is yet to be fully elucidated. Ig, immunoglobulins; p.i., pharmacological interactions; IL, interleukin; TARC, thymus and activation regulated chemokine; TNF-α, tumor necrosis factor α; IFN-γ, interferon γ.

The possible pathogenesis of the DRESS syndrome is reported in Figure 1.

## 4. Clinical manifestations

DRESS syndrome is a systemic process with quite extensive clinical spectrum (30). The symptoms generally begin 2–8 weeks after drug introduction, may persist for several weeks after drug suspension, and may cause a significant diagnostic challenge. In children, the most common clinical presentation is fever, a maculo-papular rash, and a variable degree of lymphadenopathy with associated eosinophilia, which is common but may be absent; additionally, prodromal symptoms mimicking mild viral infections may be present (4, 6). The current literature does not show significant differences in the clinical presentation between children and adults (34).

The fever usually ranges from 38 to 40°C. The maculo-papular rash is the most common initial cutaneous manifestation, but less frequently the rash is polymorphic, including purpuric, targetoid, and eczema-like lesions, blisters, and pustules (8). An erythrodermic rash with skin detachment has also been reported (37). The skin eruption has a cranio-caudal progression, is associated with facial edema, and subsequently spreads to the trunk and lower extremities (11). Mucosal involvement, such as conjunctivitis, oral, and/or genital mucositis, can occur as well. These symptoms can persist for months after discontinuation of the causative medicine (32).

The syndrome may produce damage to other organs with eosinophilic infiltration. The liver is often affected, manifesting as hepatitis, but it is not uncommon for the kidneys, lungs, and spleen to be involved as well. Clinical manifestations overlap between children and adults, despite gastrointestinal involvement being more frequent in children (11, 38). The most frequent gastrointestinal manifestations other than hepatitis are non-specific colitis and gastroenteritis with or without electrolyte abnormalities. Other gastrointestinal complications include chronic protein-losing enteropathy and pancreatitis (39). A review conducted by Metterle et al. (1) on the pediatric DRESS syndrome identified a slightly increased splenic involvement in children compared to adults, which requires further studies to be confirmed and to verify whether this may lead to an increased risk for long-term encapsulated bacterial infections. On the contrary, pulmonary involvement, such as interstitial pneumonitis, pleural effusion, pneumonia, pulmonary nodules, and acute respiratory distress syndrome, which is a rare complication of the DRESS syndrome (40), seems to be less frequent in children than in adults (11). In the series reported by Mori et al. (11), in the pediatric DRESS syndrome, hepatic involvement is present in 51–84% of cases, renal involvement in a highly variable percentage between 11 and 57%, and gastrointestinal tract involvement in 8% of cases, finally pulmonary involvement is present in 2.6–5% of cases.

Although the DRESS syndrome typically occurs 2–8 weeks after the beginning of the drug treatment, some authors report a possible span of 5 days to 16 weeks (41). A recent report of 49 French pediatric cases describes a rapid-onset DRESS syndrome, in which the temporal latency is less than 2 weeks after beginning drug use (8); these cases were most often triggered by antibiotics and iodized contrast media. In these patients, lymphadenopathy may be more frequent, but no differences have been reported concerning severity, visceral involvement, resolution, RegiSCAR score, or mortality (4). It has been hypothesized that past exposure to related drugs may account for the shorter period between drug intake and the reaction (42). As suggested by Soria et al. (43), the DRESS syndrome would then correspond to the phase in which hypersensitivity to a drug is demonstrated in previously sensitized but unaware patients or to rechallenge with a more rapid onset of a more severe cutaneous adverse drug reaction. The main differences and similarities between rapid- and delayed-onset DRESS syndrome are reported in Table 1.

**Table 1:** Comparison between rapid- and delayed-onset Drug Rash with Eosinophilia and Systemic Symptoms (DRESS) syndrome.

	Rapid-onset dress syndrome	Delayed-onset dress syndrome
Onset of symptoms	<15 days after drug initiation	>15 days after drug initiation (usually 2–8 weeks, described up to 16 weeks)
Culprit drugs (most frequent)	Antibiotics and iodinated contrast media	Anticonvulsants
Frequency	Less frequent in general, but more frequent in pediatric patients	The most frequent
Severity	No differences	No differences
Visceral involvement	No differences	No differences
Resolution	No differences	No differences
RegiSCAR score	No differences	No differences

RegiSCAR, Registry of Severe Cutaneous Adverse Reactions (33).

**Figure 2:**
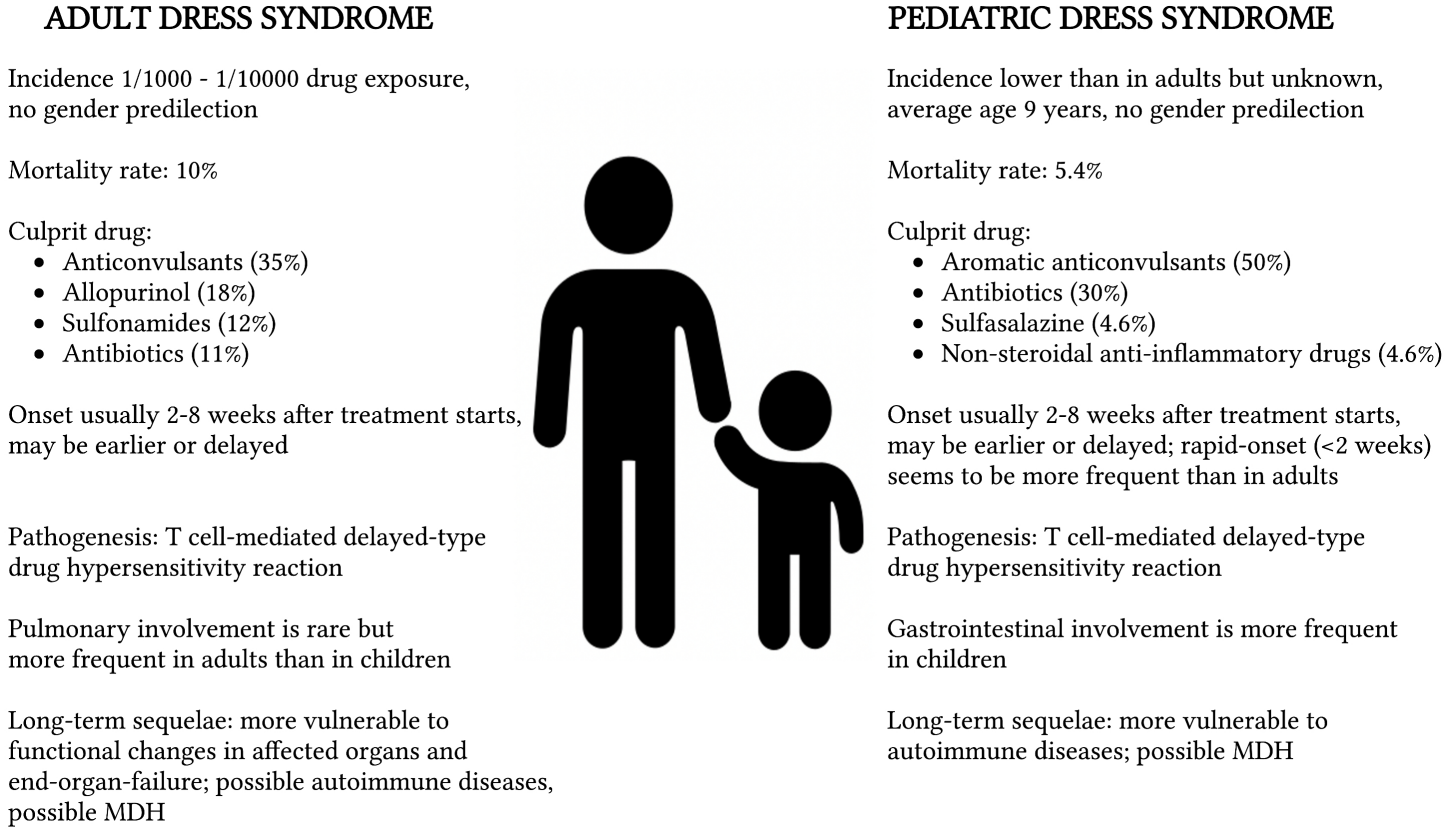
Comparison between adult and pediatric Drug Rash with Eosinophilia and Systemic Symptoms (DRESS) syndrome with reference to epidemiology (11), mortality rate (4), culprit drug (1, 19), onset of symptoms (8), pathogenesis (11), clinical manifestations (11, 38), and long-term sequelae (9, 44). MDH, Multiple Drug Hypersensitivity.

Overall, the mortality rate appears to be lower in the pediatric than in the adult population, but fatal complications, such as sepsis, fulminant hepatitis, and acute respiratory distress syndrome, may occur as well and should be monitored during follow-up (37). The comparison between adult and pediatric DRESS syndrome is described in Figure 2.

The HHV-6 reactivation is usually detected between 2 and 4 weeks after symptoms onset (22), and, in children, it is related and probably contributes to a more serious disease course, with a greater degree of systemic involvement, in particular pulmonary complications (14). Furthermore, in the DRESS syndrome, the detection of HHV-6 DNA has also been linked with flare-ups of symptoms (45).

## 5. Diagnosis

DRESS syndrome’s heterogeneous presentation and the asymptomatic period between taking the drug and the onset of symptoms make diagnosis challenging, especially in the early phases (43, 46, 47). Currently, the diagnostic criteria for DRESS syndrome are the same in children and adults and encompass the European RegiSCAR (33), the Bocquet et al.’s (6), and the Japanese group of Severe Cutaneous Adverse Reactions to Drugs (J-SCAR) (31) criteria (Tables 2, 3). DRESS syndrome should be suspected in any child with consistent clinical manifestations and the use of drugs in a latency period compatible with the DRESS syndrome; suspicion must be higher if the drug taken is one known to induce DRESS syndrome and diagnostic work-up must include a detailed clinical history with full medical background and history of drug administration.

**Table 2:** The European Registry of Severe Cutaneous Adverse Reactions (RegiSCAR) scoring system for diagnosing DRESS syndrome (33): a diagnosis of DRESS syndrome is definite (score > 5), probable (score 4–5), possible (score 2–3), or ruled out (score < 2) according to the score obtained.

Criteria	Score			
	−1	0	+1	+2
Fever ≥ 38.5°C (core) or ≥ 38°C (axillary)	No/unknown	Yes		
Enlarged lymph nodes (≥2 Sites, >1 cm)		No/unknown	Yes	
**Eosinophilia**				
Eosinophils		No/unknown	700–1,490	≥1,500
Eosinophils, if leukocytes <4 × 109/L		No/unknown	10.0–19.9%	≥20%
Atypical lymphocytes		No/unknown	Yes	
**Skin involvement**				
Skin rash extent (>50% BSA)		No/unknown	Yes	
Skin rash suggesting dress^a^	No	Unknown	Yes	
Biopsy suggesting dress	No	Yes/unknown		
**Organ involvement** ^b^				
One		No/unknown	Yes	
Two or more		No/unknown		Yes
Resolution ≥15 days	No/unknown	Yes		
At least 3 biological negative investigations done^c^ to exclude other potential diagnosis		No/unknown	Yes	

a Maculopapular rash and two of the following: (I) facial edema; (II) psoriasiform desquamation; (III) infiltrated skin lesions; (IV) purpuric lesions involving areas other than legs.

b After exclusion of other explanations.

c Investigations suggested by Kardaun et al. (19): Antinuclear antibody, blood culture, serology for hepatitis A virus, hepatitis B virus, hepatitis C virus, Chlamydia/Mycoplasma.

BSA, Body Surface Area. Total score: minimum −4, maximum 9; <2 no case, 2–3 possible case, 4–5 probable case, >5 definite case.

**Table 3:** Comparison among the three proposed criteria for the diagnosis of the Drug Rash with Eosinophilia and Systemic Symptoms (DRESS) syndrome or Drug Induced Hypersensitivity Syndrome (DIHS).

	Bocquet et al. (6)	J-SCAR (31)	RegiSCAR (19, 48)
Skin eruption	Skin drug eruption	Maculopapular rash developing at least 3 weeks after starting a suspected drug	Acute skin rash
Fever		Fever >38°C	Fever ≥38.5°C (core) or ≥38°C (axillary)
Hematological abnormalities	Eosinophilia (≥1.5 × 10^3^/μL) or presence of atypical lymphocytes	Leukocytosis (>11 × 10^3^/μL), atypical lymphocytosis (>5%) or eosinophilia (>1.5 × 10^3^/μL)	Lymphocytosis (>4 × 10^3^/μL) or lymphocytopenia (<1.5 × 10^3^/μL), blood eosinophilia (>10%), thrombocytopenia (<120 × 10^3^/μL)
Lymphadenopathy		Lymphadenopathy	Lymphadenopathy that involves ≥2 different sites
Systemic involvement	Internal organ involvement, including lymphadenopathies, hepatitis (liver transaminases values ≥2 times the upper normal limit), interstitial nephritis, interstitial pneumonia or carditis	Liver abnormalities (ALT >100 U/L) or other organs involvement	Involvement ≥1 internal organ
Other		Clinical symptoms persisting 2 weeks after discontinuing the culprit drug. HHV-6 reactivation	

Bocquet’s criteria: DRESS syndrome can be diagnosed when all 3 criteria are met. J-SCAR criteria: typical DIHS can be diagnosed when all 7 criteria are met; in case HHV-6 reactivation is absent, atypical DIHS should be considered. Registry of Severe Cutaneous Adverse Reactions (RegiSCAR) inclusion criteria: in patients hospitalized for a suspected cutaneous adverse drug reaction, a diagnosis of DRESS syndrome should be considered when at least three of the reported items are present; patients can subsequently classified into definite (score > 5), probable (score 4–5), or possible (score 2–3) cases, or ruled out (score < 2), according to the scoring system reported in Table 2.

The diagnostic criteria proposed by Bocquet et al. (6) include 3 features: (1) typical skin rash; (2) hematological alterations, consisting of blood eosinophilia (≥1.5 × 10^3^/μL) or atypical lymphocytes; (3) internal organ involvement, encompassing lymphadenopathies, hepatitis (with liver transaminases greater than twice the upper normal limit), interstitial nephritis, interstitial pneumonia or carditis. The diagnosis of DRESS syndrome is based on the presence of all 3 criteria (6).

In Japan, dermatologists use the acronym DIHS for Drug Induced Hypersensitivity Syndrome instead of DRESS syndrome, and in 2006 they established the J-SCAR diagnostic criteria, including: (1) maculopapular rash developing at least 3 weeks after starting a group of specified drugs; (2) clinical symptoms persisting 2 weeks after discontinuing the drug; (3) body temperature above 38°C; (4) elevation of liver enzymes [alanine aminotransferase (ALT) >100 U/L] or involvement of other organs; (5) hematological abnormalities such as leukocytosis (>11 × 10^3^/μL), atypical lymphocytosis (>5%) or eosinophilia (>1.5 × 10^3^/μL); (6) lymphadenopathy; (7) and HHV-6 reactivation (31, 49). All 7 parameters should be satisfied to diagnose typical DIHS, which may represent the most severe forms of DRESS syndrome (32). Patients in whom the first five criteria are met, in the absence of HHV-6 reactivation, are classified as atypical DIHS (12, 32).

The clinical score system established in 2007 by the RegiSCAR group is probably the most used and it seems to be more accurate and comprehensive (19). The RegiSCAR inclusion criteria for a potential case of DRESS syndrome involve at least three of the following independent features: (1) skin involvement with acute rash; (2) fever above 38.5°C (core) or ≥38°C (axillary); (3) lymphadenopathy in at least two different sites; (4) involvement of at least one internal organ; (5) lymphocytosis (>4 × 10^3^/μL) or lymphocytopenia (<1.5 × 10^3^/μL); (6) blood eosinophilia (>10% or 700/μL); (7) thrombocytopenia (<120 × 10^3^/μL). The diagnosis of DRESS syndrome is determined by clinical and laboratory features according to the RegiSCAR validation criteria (Table 2), with the individuation of a causal drug often reinforcing the clinical diagnosis (37). In these criteria each feature is scored, and according to the total score, patients are classified as definite (score > 5), probable (score 4–5), possible (score 2–3), or no DRESS case (score < 2) (Table 2) (19, 33, 49). Differently from the J-SCAR criteria, the RegiSCAR does not include HHV-6 reactivation, as this feature may be absent in patients with DRESS syndrome.

The lately published Spanish guidelines for DRESS syndrome suggest using the RegiSCAR criteria for clinical diagnosis (39). However, the comparative retrospective analysis conducted by Kim et al. (49) among the three criteria established that, although these criteria are the most accurate, Bocquet’s criteria seem to be the easiest to use in the clinical setting; the J-SCAR criteria are potentially restrictive and may miss episodes of DRESS syndrome otherwise recognized by the other two (49).

In patients evaluated for DRESS syndrome, laboratory tests should be performed, including a complete blood count, liver enzymes, blood electrolytes, renal function test, urine analysis, and baseline thyroid function tests (30, 50). The latter should be repeated after 2 months since drug-induced hypothyroidism could occur later. The autoimmune complications, such as type 1 diabetes mellitus and autoimmune thyroiditis, may appear with a longer latency (months or years) after the acute stage (34, 51). Moreover, the diagnostic work-up should include HHV6-, HHV7-, CMV-, and EBV-polymerase chain reaction (PCR), and serology for hepatitis A, B, and C viruses; chlamydia and mycoplasma IgM or PCR may also be performed. A skin biopsy can also be included, in front of a diagnostic doubt; however, although different reports have found perivascular dense lymphocytes infiltrate in the dermis and lichenoid features, the histopathological pattern of DRESS syndrome is highly variable and not pathognomonic (33).

According to the recent Spanish guidelines (39), additional tests that can be performed according to the patient’s symptoms are abdominal ultrasound, chest X-ray, electrocardiogram and echocardiography, computed tomography (CT) of the brain and neurological evaluation, pulmonary function tests and chest CT and evaluation by various specialists.

The clinical severity of DRESS syndrome and the risk of its recurrence could be related to the presence of fever, prolonged leukocytosis, facial edema, lymphadenopathy with erythroderma (10) and it is connected to visceral involvement when present (52).

Assessment of the culprit drug of DRESS syndrome is particularly challenging. Patch tests and lymphocyte transformation tests (LTTs) can be used to verify drug causality, and they are preferable to intracutaneous or provocative tests due to their higher safety profile. Patch tests are *in vivo* tests in which the suspect drug is diluted and applied to the skin; their positivity is extremely linked to the type of reaction and the investigated drug, and it depends on the concentration of the drug, because low concentrations may lead to a false negative, and high concentrations could result in reactivation. Previous studies have shown that patients with DRESS syndrome have a good percentage of patch test positivity (53, 54). A study of 71 pediatric patients performed by Yaytokgil et al. (55) showed that the sensitivity of patch tests is higher with antiepileptic drugs. Patch tests should be performed 2 to 6 months after symptoms resolution (56). The LTTs are *in vitro* tests that attempt to measure serum T cell activation when exposed to a specific drug. LTTs are useful in the diagnosis of delayed hypersensitivity reactions, although still experimental and difficult to perform, seem to be influenced by the type of reaction and drug, and they need an appropriate timing, which in the case of DRESS syndrome appears to be between 5 and 8 weeks after the onset of the rash (39, 57). A study conducted by Liccioli et al. (53) on a pediatric population demonstrated a positivity of LLTs in 75% of cases of DRESS syndrome, higher than in other series which however were performed on adult populations.

Further studies are needed to standardize the identification of the culprit drug in DRESS syndrome.

## 6. Differential diagnosis

The diagnosis of DRESS syndrome remains complex due to a non-specific presentation, and, in children, it is even more challenging due to the numerous potential differential diagnoses, including a high number of infections (EBV, CMV, Parvovirus, *Mycoplasma pneumoniae*, and Staphylococcal Scalded Skin Syndrome), rheumatologic diseases (Kawasaki disease, systemic onset juvenile idiopathic arthritis, systemic lupus erythematosus), hematologic diseases (macrophage activation syndrome, hematologic malignancies, pseudolymphoma), and dermatological diseases (Stevens–Johnson syndrome, toxic epidermal necrolysis) (33, 37, 41).

The differential diagnosis is often allowed by the patient’s anamnestic history, the clinical symptoms, the type of skin lesions, and by laboratory tests, including viral serologies, described in the diagnostic workup.

## 7. Treatment and prognosis

The treatment of pediatric DRESS syndrome is challenging since, to our knowledge, there are no randomized studies that have evaluated the use of a specific therapy, and a consensus for its management is still lacking (41). It is therefore exclusively dependent on early identification and related to the severity of clinical manifestations and organ involvement (10). The most relevant steps are early recognition of adverse drug reactions and instant suspension of the causative drug (3). Timing in the management of DRESS syndrome is essential, so the treatment should be started immediately after diagnosis, even if the results of virological tests are still incomplete (11), because a delay may be associated with worse outcomes. A child with DRESS syndrome should be managed through hospitalization, except in mild cases where they can be managed at home with close monitoring every 48 h (39). In mild forms the treatment is based on supportive measures to stabilize the patient and symptomatic therapy based on fluids, electrolytes, and nutritional support; the use of H1-antihistamines and topical preparations to alleviate the cutaneous symptoms, including corticosteroids and emollients has also been reported (11, 56, 58).

Steroids induce inhibition of interleukin-5 and eosinophilic accumulation, which are responsible for visceral organ damage and lead to a reduction in symptoms due to associated delayed hypersensitivity reactions (10). For these reasons, in moderate and severe disease, systemic corticosteroids are currently the most accepted and used treatment (59, 60), with a minimum initial dose of 1 mg/kg/day of prednisone or equivalent and slow tapering over 3–6 months after stabilization of clinical symptoms and laboratory abnormalities, because a rapid decrease in the dose can be associated with relapse (56). Currently, there is no consensus guideline on what degree of severity justifies the need for systemic corticosteroid therapy; however, the French Society of Dermatology recommends its use with a 5-fold increase in serum transaminase levels or with organ involvement (i.e., kidney, lung, or heart) (26, 61).

In cases of DRESS syndrome that do not respond to corticosteroid therapy, the use of second-line therapies should be evaluated. In some pediatric patients, high-dose intravenous immunoglobulin (IVIG) (1 g/kg/day for 2 days) in addition to systemic corticosteroids has been shown to be beneficial (62). IVIG compensate for the patient’s decreased immunoglobulin concentration, support immune defense against HHV-6 infection, and have a substantial anti-inflammatory effect (63). Their use is controversial, despite some authors report fewer adverse effects and better tolerance in children compared to adults (64, 65). At an early stage of the disease, however, IVIG are thought to accelerate rapid B cell recovery, resulting in an increase of autoantibodies production; the development of these autoantibodies could be prevented by using systemic corticosteroids, leading to a consensus that corticosteroids and IVIG should be used together in severe forms (66).

For patients with severe organ involvement who have contraindications to the use of steroids or who do not respond to corticosteroids, cyclosporine, a calcineurin inhibitor with immunosuppressive action, could also be evaluated as a second-line treatment (67, 68). Su et al. (69) recently demonstrated the efficacy of cyclosporine in 8 cases of corticosteroid-dependent DRESS syndrome. The mechanism of action is linked to the inhibition by cyclosporine of various cytokines (IL-2, IL-3, IL-4, IL-5, and TNF) implicated in the pathogenesis of DRESS syndrome; moreover, the study demonstrates that the therapy leads to a significant drop in serum TARC levels, further confirming the therapeutic efficacy. There is currently no standardization of dose and duration of therapy, which appears to be a promising option as a corticosteroid-sparing alternative (69).

In cases of severe DRESS syndrome unresponsive to steroid therapy, another therapeutic option to be evaluated is plasma exchange, which contributes to the reduction in circulating cytokine levels. There are cases in the literature where plasma exchange has been successfully used in pediatric patients with corticosteroid-resistant DRESS syndrome (70, 71), however, further investigation and more scientific evidence are needed (71).

Since IL-5 is implicated in the pathogenetic mechanism of DRESS syndrome (72), its axis could be ideal for targeted therapy. For this reason, research is focusing on the possible use of drugs blocking IL-5 and/or its receptor, namely mepolizumab and reslizumab, monoclonal antibodies active against IL-5, and benralizumab, active against the IL-5 receptor (73). Studies are promising and the treatment seems to have a good safety profile and no immunosuppressive effects, but the evidence is still limited, and the use of these drugs is not standardized. They are reserved for patients with refractory DRESS syndrome, severe relapse, or severe organ involvement. Moreover, the limited cases reported in the literature currently concern only adult patients with DRESS syndrome and to our knowledge currently, there is no evidence of use in children.

Antibiotics and anti-inflammatory drugs with potential cross-reactivity to the culprit drugs should not be used empirically during the acute period because they can complicate or worsen patients’ symptoms due to cross-reactivity (31).

**Figure 3:**
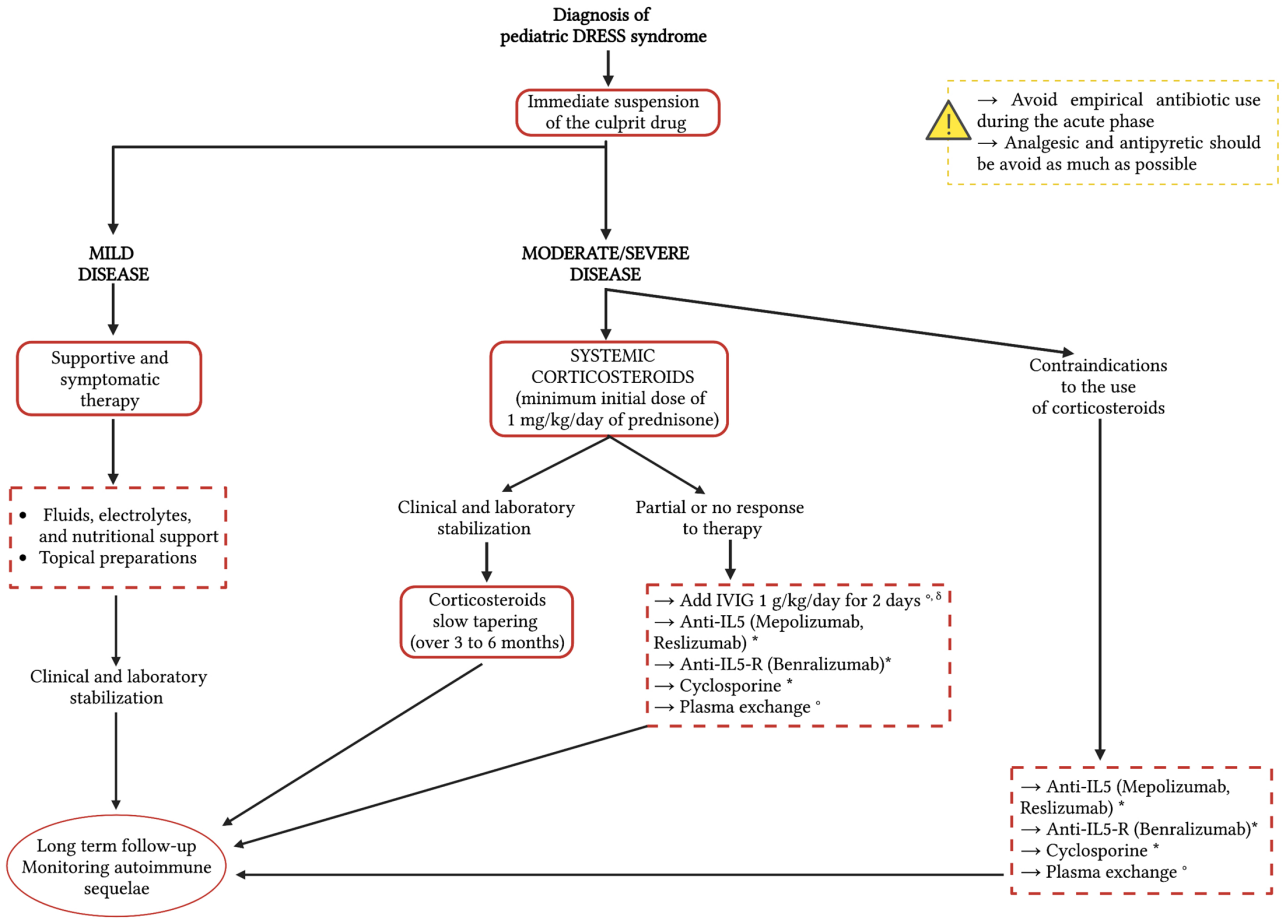
A therapeutic approach to the Drug Rash with Eosinophilia and Systemic Symptoms (DRESS) syndrome based on current scientific evidence. The red dotted line indicates possible therapeutic options, on which further studies and evidence are needed. Since there is no consensus on the therapeutic treatment of pediatric DRESS syndrome, the therapeutic options in the dotted boxes are listed in alphabetical order. * Studies available in the literature currently concern only adult patients with DRESS syndrome. ° Studies available in the literature regarding pediatric DRESS syndrome. ^δ^ Corticosteroids slow tapering (over 3–6 months). IVIG, intravenous immunoglobulins; IL-5, interleukin-5; IL-5-R, IL-5 receptor.

A therapeutic approach based on current scientific evidence is schematized in Figure 3.

After the suspension of the culprit drug, complete recovery is not immediate but takes a few weeks for most patients. According to a literature review, DRESS syndrome recurrence in the pediatric population appears to be strongly related to the presence of fever, facial edema, lymph node enlargement, leukocytosis, pharyngeal and internal organ involvement, and chronic medical conditions (10). The course of the DRESS syndrome frequently shows various episodes of complete or partial recurrences, despite removing the culprit drug. Reactivation of cutaneous manifestations during the first months after the episode often occurs in a milder form and in a shorter time window after drug re-exposure. Relapses are more frequent in children who have comorbidities, including neuropsychiatric conditions, and who require long-term therapy with anticonvulsants, which could induce a new episode due to cross-reactivity between some molecules (10).

Long-term sequelae are described in 10.8% of the pediatric population and are defined as conditions observed 1–24 months after the DRESS syndrome; the most common are autoimmune diseases, including hypothyroidism, type 1 diabetes mellitus, systemic lupus erythematosus, systemic sclerosis, adrenal insufficiency, and autoimmune hemolytic anemia, which can occur months to years after the resolution of the syndrome, highlighting the importance of long-term follow-up and surveillance (1, 9, 44, 74). Autoimmune sequelae are more common in young people, while older patients are more vulnerable to organ failure (44). A review conducted by Tempark et al. (75), showed a variable onset of thyroid dysfunction after DRESS syndrome, with a median of 2 months in children and 6 months in adults.

A recent study demonstrated that interferon-γ -induced protein (IP)-10 is associated with HHV-6 reactivation and a higher incidence of long-term sequelae, which is why in the future it could be used as a predictive marker for the development of sequelae (76). Previous studies have shown that children have an increased risk of developing type 3 polyglandular autoimmune syndrome as a sequela of DRESS syndrome. It has therefore been suggested to subject patients with DRESS syndrome, especially children, to an autoantibody screening and in patients at risk to carefully evaluate whether to subject them to therapies that induce a rapid increase in B and T cells, such as IVIG and pulsed prednisolone (77).

MDH is often seen as a complication of DRESS syndrome, related not to the disease itself but more to the strong T-cell stimulation. Moreover, patients with DRESS syndrome represent a high-risk group for developing MDH, so they need to be monitored when new drugs are administered (23).

## 8. Conclusion

The DRESS syndrome is a rare but severe drug hypersensitivity reaction characterized by systemic inflammation and multisystem involvement. A consensus on diagnosis and terminology is lacking. Although it is mainly triggered by anticonvulsants, in the pediatric age group, the rate of cases secondary to antibiotics is much higher than in adults. Recognizing the DRESS syndrome is challenging, and this often leads to a delay in diagnosis and the discontinuation of the causative drug. Knowing the syndrome is essential to be able to diagnose it since its signs and symptoms often evolve sequentially. Pediatric DRESS syndrome should be supposed when a child presents with fever, maculopapular eruption, lymphadenopathy, eosinophilia, and visceral involvement. The onset of the symptoms may be delayed (2–6 weeks) or rapid (<15 days). Quick identification and prompt suspension of the culprit drug are the most decisive actions to avoid syndrome progression. More research is required to better explain the clinical implications and set treatment standards for pediatric DRESS syndrome.

## Author contributions

AD: conception of the work, manuscript draft, and critical revision. EM: conception of the work and first manuscript draft. IN and ML: critical revision for important intellectual content. All authors provide approval for publication of the content and agree to be accountable for all aspects of the work in ensuring that questions related to the accuracy or integrity of any part of the work are appropriately investigated and resolved.

## Conflict of interest

The authors declare that the research was conducted in the absence of any commercial or financial relationships that could be construed as a potential conflict of interest.

## Publisher’s note

All claims expressed in this article are solely those of the authors and do not necessarily represent those of their affiliated organizations, or those of the publisher, the editors and the reviewers. Any product that may be evaluated in this article, or claim that may be made by its manufacturer, is not guaranteed or endorsed by the publisher.
